# Bilateral optic perineuritis with vestibulocochlear involvement: revisiting a rarely performed biopsy

**DOI:** 10.17879/freeneuropathology-2026-9407

**Published:** 2026-04-09

**Authors:** Katrina J. Jiang, Marianne E. Yassa, Saad Awan, Osama R. Elkadi

**Affiliations:** 1 Department of Pathology, University of South Alabama University Hospital, Mobile, Alabama, USA; 2 Department of Radiology, University of South Alabama University Hospital, Mobile, Alabama, USA

**Keywords:** Optic perineuritis, Optic neuritis

## Letter

Bilateral optic perineuritis (OPN) is an uncommon inflammatory disorder of the optic nerve sheaths typically distinguished from optic neuritis (ON) by perineural enhancement, relative sparing of central vision, and improvement with corticosteroids.^1,2^ While bilateral OPN can be idiopathic, it is more frequently associated with systemic inflammatory conditions or infections.^3^ Histopathologic characterization is limited, as most published cases are diagnosed radiographically and clinically. We present a case of treatment-refractory bilateral OPN with vestibulocochlear involvement, in which a dural biopsy revealed a lymphocytic inflammatory infiltrate without granulomatous, infectious, or neoplastic features. 

**Figure 1:**
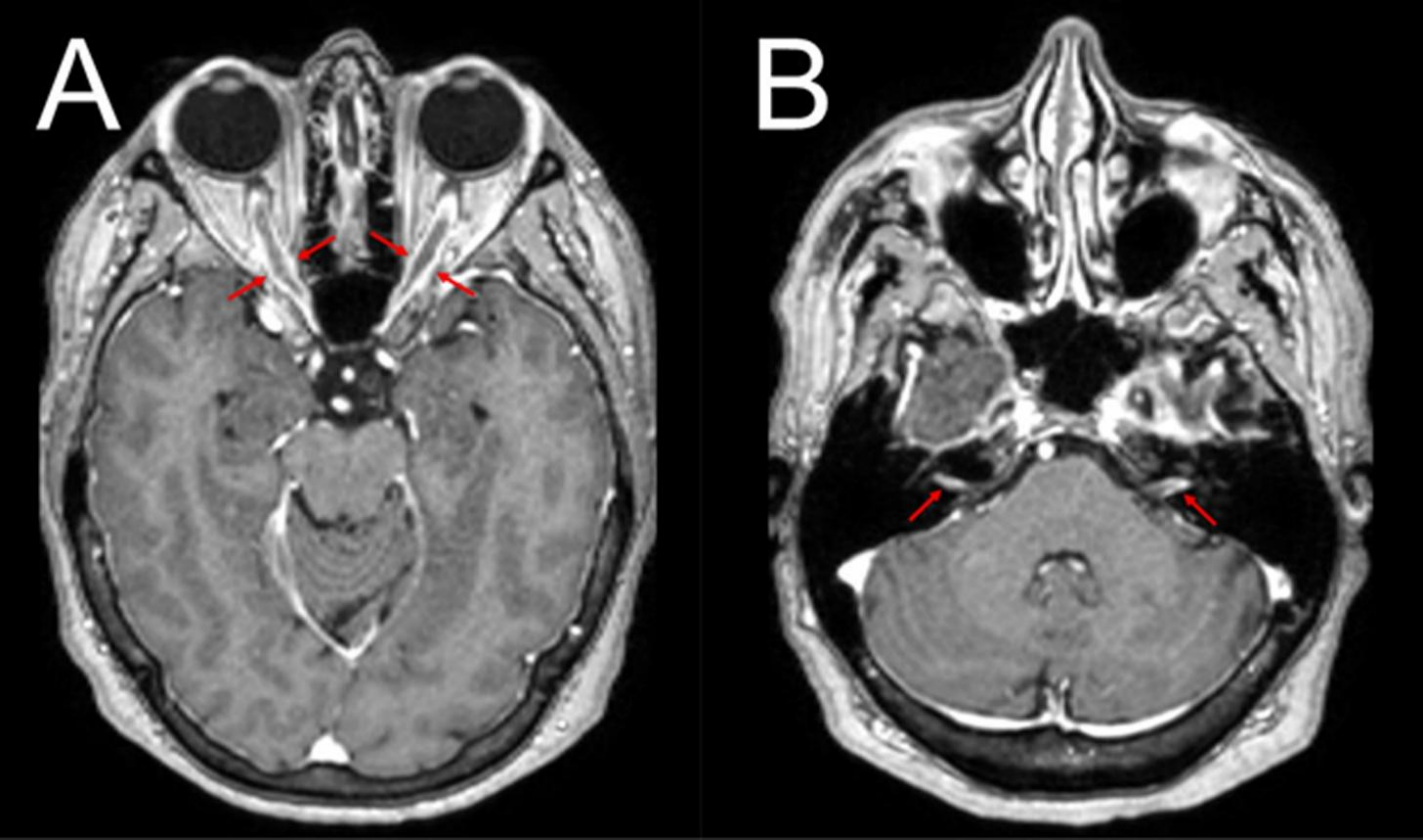
**A.** Orbit MRI (post-contrast axial T1-weighted) revealed circumferential thickening and enhancement of the bilateral optic nerve sheaths (red arrows). **B.** The same MRI showed circumferential left-greater-than-right vestibulocochlear nerve enhancement (red arrows).

A 68-year-old female with well-controlled hypertension, recurrent urinary tract infections (UTIs), and no history of autoimmune disease presented with progressive, bilateral, painless vision loss, headaches, and bilateral hearing loss. Symptoms began in the left eye before affecting the right eye three months later. Central vision was lost bilaterally, while peripheral vision was only initially preserved in the right eye. The pupillary light reflex on the left was absent, and that on the right was markedly weak. Fundoscopy was normal without optic disc edema or pallor. MRI of the brain and orbits showed circumferential thickening and enhancement of the bilateral optic nerve sheaths, along with left-greater-than-right vestibulocochlear nerve enhancement (**Figure 1**). Cerebrospinal fluid (CSF) and serum studies were negative for infectious, paraneoplastic, demyelinating, and autoimmune markers, including myelin oligodendrocyte glycoprotein immunoglobulin G (MOG-IgG) and aquaporin4-IgG, with only a non-specific IL-2 elevation in CSF (31.4 pg/mL). Positron emission tomography/computed tomography was negative. The patient only partially improved with corticosteroids and was unresponsive to plasma exchange, rituximab, infliximab, and intravenous immunoglobulin.

**Figure 2:**
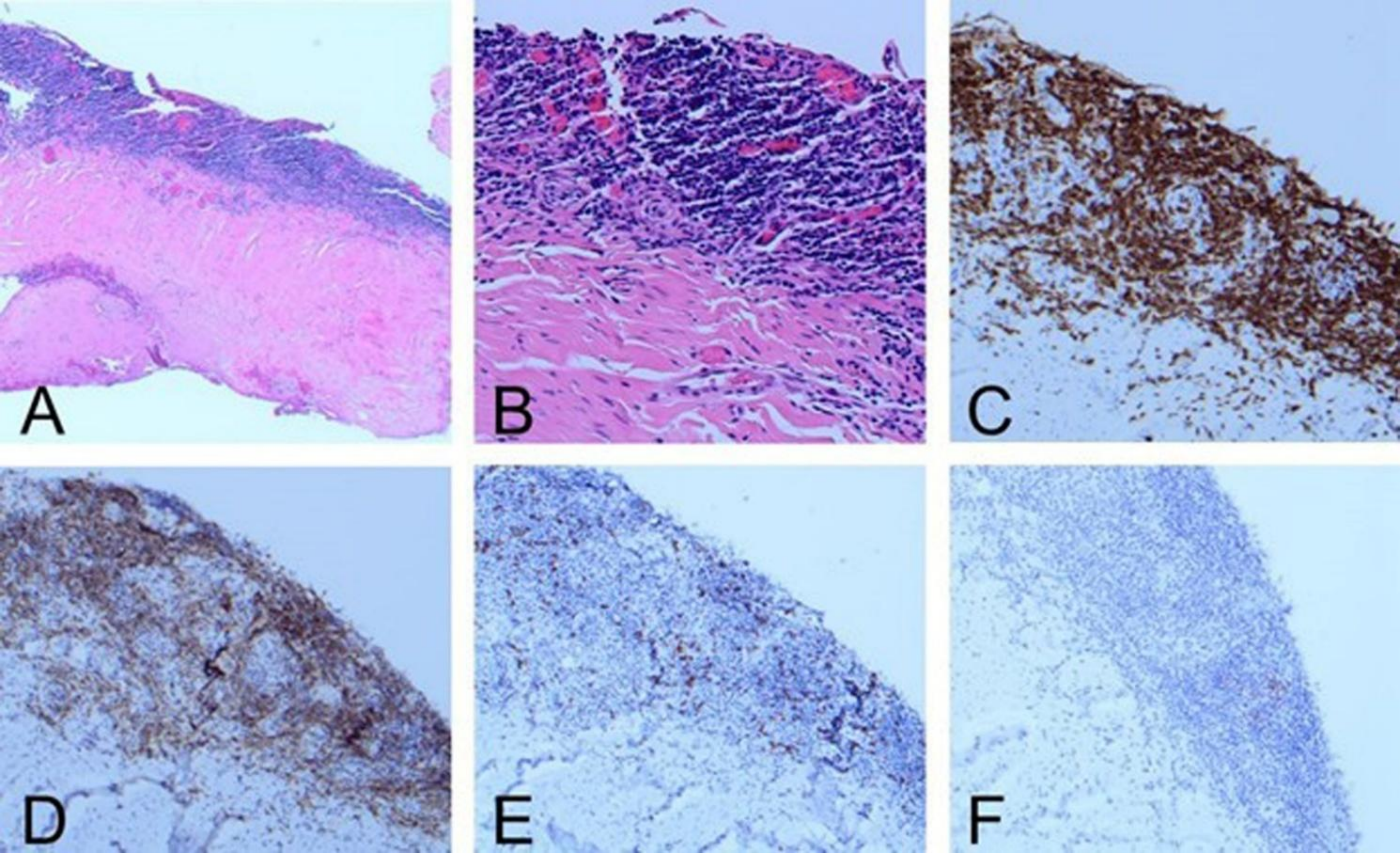
**A, B.** Band-like lymphocytic infiltrate along the subdural surface (**A:** H&E, 4X; **B:** H&E, 20X). **C. **Infiltrate composed predominantly of small CD3-positive T-cells (**C:** CD3 IHC, 10X). **D, E.** Normal CD4:CD8 ratio (**D:** CD4 IHC, 10X; **E: **CD8 IHC, 10X). **F.** Markedly decreased CD20 expression consistent with rituximab exposure (**F:** CD20 IHC, 10X).

Given the lack of therapeutic response, a left mini-pterional craniotomy with temporal dural excisional biopsy was performed. Two dusky pink-tan fibromembranous tissue fragments measuring 1.0 x 0.6 x 0.2 cm and 1.0 x 0.5 x 0.2 cm were submitted for pathologic evaluation. Hematoxylin and eosin (H&E)-stained sections revealed a band-like lymphocytic infiltrate along the subdural surface, which immunohistochemistry (IHC) showed to be composed predominantly of small CD3-positive T-cells with a normal CD4:CD8 ratio (**Figure 2**) and polytypic TRBC1 expression by multiplex staining. There was associated vascular and reactive meningothelial proliferation with fibrosis. Small PAX5-positive B-cell aggregates showed markedly decreased CD20 expression, consistent with recent rituximab exposure. CD138-positive plasma cells were rare and polytypic, and immunostains for IgG and IgG4 were negative. Scattered CD68-positive histiocytes did not show abnormal cyclin D1 staining. Additional stains (including HSV, CMV, and PAS/D) showed no granulomas, vasculitis, malignancy, viral infection, or other infectious organisms. Given the absence of systemic disease, the findings favored idiopathic inflammatory OPN.

Our patient’s histology aligns with prior microscopic descriptions of OPN, which typically describe a predominantly lymphocytic infiltration of the optic nerve sheath along with non-specific fibrosis.^2^ It is theorized that the thickened optic nerve sheath circumferentially compresses the optic nerve, causing ischemic injury and vision loss. There have also been rare reports of necrobiotic collagen, vasculitic changes of the small optic nerve vessels, or granulomatous inflammation,^2^ though these features were absent in our case. Few modern cases include immunophenotyping or clonality studies, which were performed in our workup.

OPN is frequently associated with systemic inflammatory conditions, especially in bilateral presentations. A Canadian series found the most common secondary etiologies of OPN were myelin oligodendrocyte glycoprotein antibody-associated disease, syphilis, sarcoidosis, and giant cell arteritis.^4^ Notably, idiopathic cases in the series were all unilateral presentations. A Chinese cohort study reported nearly one quarter of OPN cases were MOG-IgG seropositive, and these patients tended to be younger, more prone to relapse, and more responsive to corticosteroids.^5^ Our case is unusual, as it demonstrates additional cranial nerve involvement of the bilateral vestibulocochlear nerves, a finding reported only once previously in a sinus biopsy-proven IgG4-related bilateral OPN with bilateral maxillary nerve involvement.^6^ This report was one of only three biopsy-proven IgG4-related OPNs in the literature,^6^ highlighting again the lack of histopathologic characterization of this disease. In our case, the patient was MOG-IgG seronegative, and her biopsy was negative for IgG4 staining. These findings support an idiopathic etiology.

Other infectious and secondary causes of bilateral OPN, including syphilis^9^ and tuberculosis,^10^ have been reported. Bilateral OPN was also reported in an ICU patient with COVID-19, supporting a cytokine-mediated inflammatory mechanism.^11^ Bilateral OPN has also been described in two obese female patients with papilledema: one with idiopathic intracranial hypertension (IIH) confirmed by magnetic resonance imaging (MRI);^12^ and another with idiopathic bilateral OPN mimicking IIH clinically, as the latter was not detected by MRI.^13^ An additional idiopathic bilateral OPN case was reported in a 64-year-old male with hypertension. He showed complete recovery with corticosteroids, although biopsy was not performed.^14^

Following biopsy, our patient was initiated on combined corticosteroid and cyclophosphamide therapy, which was selected based on the biopsy’s lymphocytic profile and the lymphocyte-targeting mechanistic action of cyclophosphamide. Over the subsequent two months, she completed five cycles of cyclophosphamide with minimal initial improvement before again becoming refractory with continued waxing and waning of both vision and hearing deficits. Tocilizumab, an IL-6-targeting biologic, is planned as the next therapeutic trial. Cogan syndrome, a rare autoimmune disorder of unclear pathogenesis affecting ocular, inner-ear, and vascular tissues,^15^ became the leading differential diagnosis given the patient’s refractory bilateral optic and vestibulocochlear nerve involvement. Although our patient did not have the classically associated keratitis or iritis, she later developed intermittent ocular injection, a finding reported in most Cogan syndrome patients. Histopathologically, Cogan syndrome is characterized by a chronic inflammatory process with lymphoplasmacytic infiltration, vascular inflammation, and fibroblast proliferation. Currently, no specific antibody has been identified as a definitive diagnostic marker.^15^ Because Cogan syndrome is a diagnosis of exclusion, further evaluation is ongoing to rule out alternative etiologies in our patient.

In summary, this case provides neuropathologic characterization of bilateral OPN, demonstrating chronic, polytypic T-cell–predominant dural inflammation corresponding to radiologic involvement of both optic and vestibulocochlear nerves. Biopsy offers significant diagnostic value in treatment-refractory, seronegative, or multi-cranial nerve presentations by evaluating for underlying processes including IgG4-related disease, granulomatous inflammation, vasculitis, and malignancy. Microscopic confirmation of OPN with biopsy material often also involves sampling of the optic nerve sheath, a procedure associated with significant morbidity, particularly when the affected eye retains vision.^16^ Sampling of the optic nerve sheath carries a diagnostic yield of only 55 %.^17^ Together, these limitations explain why histopathologic confirmation remains uncommon in OPN and why most published cases rely on radiographic and clinical impressions. In our patient, a craniotomy with temporal dural biopsy provided diagnostic tissue and avoided the morbidity associated with a direct optic nerve sheath biopsy.

## Conflict of interest statement

All authors have reviewed and approved the submitted manuscript and declare no conflicts of interest related to this work.

## References

[R1] Purvin V, Kawasaki A, Jacobson DM. Optic Perineuritis: Clinical and Radiographic Features. Arch Ophthalmol. 2001;119(9):1299-1306. 10.1001/archopht.119.9.1299 11545635

[R2] Gupta S, Sethi P, Duvesh R, Sethi HS, Naik M, Rai HK. Optic perineuritis. BMJ Open Ophthalmol. 2021;6(1):e000745. 10.1136/bmjophth-2021-000745 PMC8144033 34104798

[R3] Miller C, Vu NH, Carozza RB. Idiopathic Optic Perineuritis in a Pediatric Patient. J Child Neurol. 2025;40(10):906-909. 10.1177/08830738251341771 PMC12508487 40415402

[R4] Xie JS, Donaldson L, Margolin E. Optic perineuritis: A Canadian case series and literature review. J Neurol Sci. 2021;430:120035. 10.1016/j.jns.2021.120035 34717266

[R5] Cao S, Zhang Y, Xu X, et al. Antibodies to myelin oligodendrocyte glycoprotein in optic perineuritis. Front Immunol. 2025;16:1657600. 10.3389/fimmu.2025.1657600 PMC12605376 41235231

[R6] Hung CH, Lo CY. Immunoglobulin G4-Related Orbital Disease with Bilateral Optic Perineuritis and Maxillary Nerves Involvement: A Case Report. Ophthalmol Ther. 2020;9(4):1089-1099. 10.1007/s40123-020-00313-2 PMC7708560 33068267

[R7] Lemaitre S, Esquerda GM, Guardiola AC, Agustin JT, Sanda N, González-Candial M. Colon cancer and IgG4-related disease with orbital inflammation and bilateral optic perineuritis. Medicine (Baltimore). 2018;97(39):e12197. 10.1097/MD.0000000000012197 PMC6181517 30278491

[R8] Lee CS, Harocopos GJ, Kraus CL, et al. IgG4-associated orbital and ocular inflammation. J Ophthalmic Inflamm Infect. 2015;5(1):15. 10.1186/s12348-015-0047-y PMC4446498 26034515

[R9] Feemster JC, Browne JD, Green KE. Bilateral Optic Perineuritis Secondary to Treponema pallidum Infection. Ophthalmology. 2025;132(8):e151. 10.1016/j.ophtha.2024.11.015 39674937

[R10] Ismail MA, Shariffudin NS, Bt Abd Jalil NF, Yew TC, Wan Hitam WH. Concurrent Tuberculous Optic Neuritis and Optic Perineuritis in a Patient With Human Immunodeficiency Virus (HIV). Cureus . 16(3):e55867. 10.7759/cureus.55867 PMC11002712 38595896

[R11] Ali L, Naeem M, Canibano B, John A, Iqrar A. Bilateral Acute Optic Perineuritis Associated With COVID-19 in a Patient With Seronegative Myelin Oligodendrocyte Glycoprotein (MOG) Antibody. Cureus . 13(9):e18234. 10.7759/cureus.18234 PMC8542256 34712522

[R12] Shahrudin NFH, Muhammed J, Wan Hitam WH, Sapiai NA, Abdul Halim S. A Case Report of Bilateral Optic Perineuritis With Idiopathic Intracranial Hypertension: Challenges in Diagnosis and Management. Cureus . 16(2):e54692. 10.7759/cureus.54692 PMC10960579 38523970

[R13] Bellucci G, De Riggi M, Di Bonaventura C, et al. Blurred lines: bilateral optic perineuritis mimicking idiopathic intracranial hypertension. Neurol Sci Off J Ital Neurol Soc Ital Soc Clin Neurophysiol . 2024;45(4):1783-1785. 10.1007/s10072-023-07215-8 38006468

[R14] Thamotaran T, Ngoo QZ, Wan Hitam WH, Yaakub A, Koh YN. A Noteworthy Case of Bilateral Idiopathic Optic Perineuritis With No Perception to Light Eye. Cureus . 14(8):e28651. 10.7759/cureus.28651 PMC9525245 36196315

[R15] Bowers E, Tripathy K. Cogan Syndrome. In: StatPearls . StatPearls Publishing; 2026. Accessed March 29, 2026. 35593853

[R16] Levin MH, Ney JJ, Venneti S, et al. Optic Nerve Biopsy in the Management of Progressive Optic Neuropathy. J Neuroophthalmol. 2012;32(4):313. 10.1097/WNO.0b013e31825be81e 22684127

[R17] Coombs RA, Chen JJ, Salomão DR, et al. Optic Nerve and Optic Nerve Sheath Biopsy Indications and Outcomes. J Neuroophthalmol. 2025;45(3):267. 10.1097/WNO.0000000000002329 40369726

